# Sphincter-sparing surgery after preoperative radiotherapy for low rectal cancers: feasibility, oncologic results and quality of life outcomes

**DOI:** 10.1054/bjoc.1999.1052

**Published:** 2000-02-21

**Authors:** A S Allal, S Bieri, A Pelloni, V Spataro, S Anchisi, P Ambrosetti, M A G Sprangers, J M Kurtz, P Gertsch

**Affiliations:** Division of Radiation Oncology and Department of Surgery, University Hospital of Geneva, 1211 Geneva 14, Switzerland; Division of Radiation Oncology and Department of Surgery, Division of Medical Oncology, Regional Hospital of Bellinzona, Switzerland; Department of Medical Psychology, Academic Medical Center, Amsterdam, The Netherlands

**Keywords:** low rectal cancers, radiotherapy, restorative surgery

## Abstract

The present study assesses the choice of surgical procedure, oncologic results and quality of life (QOL) outcomes in a retrospective cohort of 53 patients with low-lying rectal cancers (within 6 cm of the anal verge) treated surgically following preoperative radiotherapy (RT, median dose 45 Gy) with or without concomitant 5-fluorouracil. QOL was assessed in 23 patients by using two questionnaires developed by the QOL Study Group of the European Organization for Research and Treatment of Cancer: EORTC QLQ-C30 and EORTC QLQ-CR38. After a median interval of 29 days from completion of RT, abdominoperineal resection (APR) was performed in 29 patients (55%), low anterior resection in 23 patients (20 with coloanal anastomosis) and transrectal excision in one patient. The 3-year actuarial overall survival and locoregional control rates were 71.4% and 77.5% respectively, with no differences observed between patients operated by APR or restorative procedures. For all scales of EORTC QLQ-C30 and EORTC QLQ-CR38, no significant differences in median scores were observed between the two surgical groups. Although patients having had APR tended to report a lower body image score (*P* = 0.12) and more sexual dysfunction in male patients, all APR patients tended to report better physical function, future perspective and global QOL. In conclusion, sphincter-sparing surgery after preoperative RT seems to be feasible, in routine practice, in a significant proportion of low rectal cancers without compromising the oncologic results. However, prospective studies are mandatory to confirm this finding and to clarify the putative QOL advantages of sphincter-conserving approaches. © 2000 Cancer Research Campaign


*British Journal of Cancer* (2000) **82**(6), 1131–1137
© 2000 Cancer Research Campaign
Article no. bjoc.1999.1052, available online at http://www.idealibrary.com on IDEAL®

### Sphincter-sparing surgery after preoperative radiotherapy for low rectal cancers: feasibility, oncologic results and quality of life outcomes

**AS Allal[1], S Bieri[3], A Pelloni[4], V Spataro[5], S Anchisi[1], P Ambrosetti[2], MAG Sprangers[6], JM Kurtz[1] and P Gertsch[4]**

[1]Division of Radiation Oncology and [2]Department of Surgery, University Hospital of Geneva, 1211 Geneva 14, Switzerland; [3]Division of Radiation Oncology, [4]Department of Surgery and [5]Division of Medical Oncology, Regional Hospital of Bellinzona, Switzerland; [6]Department of Medical Psychology, Academic Medical Center, Amsterdam, The Netherlands

**Summary** The present study assesses the choice of surgical procedure, oncologic results and quality of life (QOL) outcomes in a retrospective cohort of 53 patients with low-lying rectal cancers (within 6 cm of the anal verge) treated surgically following preoperative radiotherapy (RT, median dose 45 Gy) with or without concomitant 5-fluorouracil. QOL was assessed in 23 patients by using two questionnaires developed by the QOL Study Group of the European Organization for Research and Treatment of Cancer: EORTC QLQ-C30 and EORTC QLQ-CR38. After a median interval of 29 days from completion of RT, abdominoperineal resection (APR) was performed in 29 patients (55%), low anterior resection in 23 patients (20 with coloanal anastomosis) and transrectal excision in one patient. The 3-year actuarial overall survival and locoregional control rates were 71.4% and 77.5% respectively, with no differences observed between patients operated by APR or restorative procedures. For all scales of EORTC QLQ-C30 and EORTC QLQ-CR38, no significant differences in median scores were observed between the two surgical groups. Although patients having had APR tended to report a lower body image score ($P$ = 0.12) and more sexual dysfunction in male patients, all APR patients tended to report better physical function, future perspective and global QOL. In conclusion, sphincter-sparing surgery after preoperative RT seems to be feasible, in routine practice, in a significant proportion of low rectal cancers without compromising the oncologic results. However, prospective studies are mandatory to confirm this finding and to clarify the putative QOL advantages of sphincter-conserving approaches. © 2000 Cancer Research Campaign

**Keywords:** low rectal cancers; radiotherapy; restorative surgery

Cancers of the distal rectum pose the double problem of local tumour control and sphincter preservation. Abdominoperineal resection (APR), long considered the standard treatment of tumours with a distal edge located up to 6 cm from the anal verge, provides local control in a substantial majority of cases, but the resulting loss of sphincter function represents a psychological burden for many patients (MacDonald and Anderson, 1985; Billingham, 1992). Consequently there has been a resurgence of interest in sphincter-conserving surgical approaches, under the suppostition that the quality of life (QOL) of such patients should thereby be improved. A more precise understanding of failure patterns, leading to the acceptance of distal margins of less than 2 cm, and the recent progress in bowel stapling techniques have made coloanal anastomoses feasible after low rectal excision. This latter approach is often associated with preoperative radiotherapy (RT), which may be considered to compensate for the limitations of the surgical technique resulting from narrow radial and distal surgical margins (Papillon and Gerard, 1990; Marks et al, 1993; Rouanet et al, 1995).

Most reports on the feasibility and results of sphincter-conserving approaches in low rectal cancers originate from centres with long-standing research interest in this area. However, there are few data concerning the applicability of these techniques in a routine general hospital surgical practice. The aims of the present study were, first, to assess retrospectively the choice of surgical procedure and the results obtained in a contemporary series of patients presenting with low-lying rectal cancers who were treated with preoperative RT in two Swiss general hospitals. The second was to formally assess QOL outcomes in patients treated with restorative procedures, compared with those in patients after APR.

*Received 15 February 1999*
*Revised 21 September 1999*
*Accepted 20 October 1999*

*Correspondence to:* AS Allal

### METHODS

### Patients

From November 1991 to September 1997, 53 consecutive patients with primary cancers involving the distal rectum (up to 6 cm or less from the anal verge) were treated by preoperative RT, with or without concomitant chemotherapy, at the University Hospital of Geneva or the Regional Hospital of Bellinzona. The aim of the preoperative RT was essentially to achieve down-staging in T2 tumours, while for T3–T4 tumours the aim was both improvement of local control and down-staging. All patients had a pretreatment biopsy diagnosis of adenocarcinoma (nine well-differentiated, 39 moderately differentiated, three poorly differentiated and unspecified in two cases). Pretreatment evaluation included physical examination, proctoscopy and/or colonoscopy, abdominal/pelvic computerized tomography (CT), chest X-ray and serum carcino-embryonic antigen (CEA). Transrectal ultrasound was carried out

**Table 1** Pretreatment patient characteristics (53)

| | |
|---|---|
| Age, mean (s.d.) | 64 years (9) |
| Gender, male/female | 37/16 |
| WHO performance status | |
| 0 | 37 |
| 1–2 | 16 |
| Distance from anal verge to the lower tumour edge (cm) | |
| Mean (s.d.) | 3.7 (1.5) |
| Maximum tumour dimension (cm) | |
| Mean (s.d.) | 7 (3) |
| TNM stage | |
| T2 | 5 |
| T3 | 31 |
| T4 | 17 |
| N0 | 42 |
| N1 | 11 |
| M0 | 51 |
| M1 | 1 |
| Mx | 1 |

in a minority of patients. The distance between anal verge and the caudal edge of the tumour was assessed by proctoscopy and/or digital examination. All tumours were classified according to the TNM system of the Union International Contre le Cancer (1992). The preoperative tumour classification was determined using information from the digital examination (including assessment of mobility), CT scan and/or transrectal ultrasound, when available. Pretreatment patient characteristics are displayed in Table 1.

### Preoperative therapy

All patients were treated with external megavoltage (6–18 MV) photon beams. A 3- or 4-field technique was used in most patients, except for seven patients treated with opposed antero-posterior pelvic fields. The median dose to the pelvic volume was 45 Gy in 25 fractions over 5 weeks (range 25.2–50.4 Gy). Ten patients received a boost of 5.4 Gy in 3 fractions directed to the tumour with 2–3 cm margin to attain a total tumour dose of 50.4 Gy. During RT, 30 patients received 5-fluorouracil (5-FU)-based chemotherapy, either in daily continuous administration (16 patients), or as intravenous (i.v.) bolus for 3–5 days during weeks 1 and 5 (14 patients). After surgery, patients with pathological nodal involvement or distant metastasis usually received post-operative 5-FU-based chemotherapy.

### Surgery

Surgery was performed at a median interval of 29 days (range 12–96 days) from completion of RT. While surgery was recommended to be carried out 4–6 weeks after RT, some patients were operated on either earlier or later, mainly due to patient preferences. APR was carried out in 29 patients, low anterior resection (LAR) in 23 patients (20 with coloanal and three with colorectal anastomoses) and transrectal excision in one patient. A temporary transverse colostomy was performed in 13 patients and a J colonic pouch in 14 patients. Of three patients found to have hepatic metastases at surgery, one underwent apparently complete resection of liver metastases.

In patients undergoing LAR with coloanal anastomosis, the technique was standardized according to the following steps: the left colon with the splenic flexure was mobilized after ligature and section of the inferior mesenteric vein and artery. The mesosigmoid was then mobilized and the plane of the mesorectum was entered in continuity as the dissection proceeded distally. This plane was followed down to the level of the pelvic floor when a total mesorectal excision (TME) was performed (Heald and Ryall, 1986). The dissection of the rectum and mesorectum was performed applying slight lateral traction to open the cleavage plane between the mesorectum and the lateral wall of the pelvis. This allowed clear visualization and preservation of the pelvic autonomic nerves. Most patients operated on with TME have had a defunctioning transverse colostomy.

### Anal function assessment

Anal sphincter function was evaluated according to the Memorial Sloan-Kettering Cancer Center (MSKCC) anal function criteria (Minsky et al, 1995). The score ‘excellent’ corresponds to 1–2 bowel movements per day and no soilage; ‘good’ corresponds to 3–4 bowel movements per day and/or mild soilage; ‘fair’ corresponds to more than 4 bowel movements per day and/or moderate soilage, and ‘poor’ corresponds to incontinence. Acute and late RT-related complications were classified according to the RTOG grading system (Perez and Brady, 1992).

### QOL assessment

Patients were selected for QOL assessment if they had 1-year minimum follow-up, did not present uncontrolled locoregional failure, were not under treatment for distant metastases and accepted participation in the study. QOL was assessed by using two questionnaires developed by the QOL Study Group of the European Organization for Research and Treatment of Cancer: a validated questionnaire assessing cancer-specific QOL (EORTC QLQ-C30) (Aaronson et al, 1993) and one assessing site-specific (colorectal) QOL (EORTC QLQ-CR38), which is in the process of validation.

### *EORTC QLQ-C30*

This is a patient self-rating questionnaire that comprises six function scales measuring physical, role, social, emotional and cognitive functions, and overall QOL, as well as symptom scales assessing pain, fatigue, emesis, bowel function, dyspnoea, appetite loss and sleep disturbances. A final item evaluates the perceived economic impact of the disease.

### *EORTC QLQ-CR38*

This module is a patient self-rating questionnaire that comprises 38 questions, of which 19 are completed by all patients and the remaining by subset of patients (males or females; patients with or without a stoma). The general structure comprises four multi-item/single-function scales (assessing body image, sexual functioning, sexual enjoyment and future perspective), seven symptom scales (assessing radiotherapy side-effects on micturition, chemotherapy side-effects, gastrointestinal general symptoms, defecation problems, stoma-related problems and sexual dysfunction in males or females) and one single-symptom item assessing weight loss. This module has been validated in The Netherlands and is currently being used in a wide range of cross-cultural studies (Sprangers et al, 1999).

© 2000 Cancer Research Campaign

**Table 2** Type of resection performed in relation to the procedure originally planned

| Planned surgery | No. of patients | Performed surgery | No. of patients (%) |
|---|---|---|---|
| APR | 21 | APR | 19 (90.5) |
| | | Restorative surgery | 2 (9.5) |
| Restorative surgery | 7 | APR | 3 (43) |
| | | Restorative surgery | 4 (57) |
| According to tumour response | 25 | APR | 7 (28) |
| | | Restorative surgery | 18 (72) |

APR, abdomino-perineal resection.

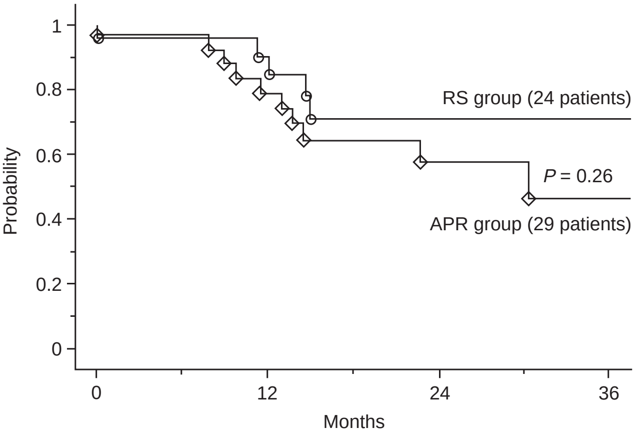

**Figure 1** Actuarial disease-free survival in the two surgical groups. APR, abdomino-perineal resection; RS: restorative surgery

### Statistical analyses

Actuarial locoregional control, overall and disease-free survival rates were calculated by the product-limit method (Kaplan and Meier, 1958). The log-rank test was used to compare survival curves, the Mann–Whitney *U*-test to compare median scores of QOL scales (considering the non-Gaussian distribution of these scores), and the Student's *t*-test to compare the means of the remaining outcome variables. A difference with a *P*-value $\leq 0.05$ was considered as significant.

All scores of the QLQ-C30 and QLQ-CR38 were linearly transformed such that all scales range from 0 to 100. The higher scale score represents a higher level of functioning for the six (QLQ-C30) and four (QLQ-CR38) multi-item/single-function scales and a higher level of symptomatology/problems for the symptom/single-item scales. We hypothesized that at least some scores of the various scales would vary between subgroups of patients according to the type of surgery, particularly levels of physical and social functions, overall quality of life, body image and sexual functioning that were expected to be higher in patients with a conserved anal sphincter.

### RESULTS

### Pathologic findings

At pathological evaluation of the resection specimen, 12 patients (22.5%) had a marked response to preoperative therapy, including four patients (7.5%) with a microscopically complete response, and eight (15%) with only microscopically residual carcinoma. The remaining 41 patients had a partial response or no change. Post-RT pathological tumour stages were four pT0, four pT1, 16 pT2, 24 pT3 and five pT4. Sixteen patients had positive lymph nodes (13 pN1 and three pN2), and three patients had hepatic metastases. All patients with positive nodes or distant metastases had partial response or no change of their primary tumour after RT. The mean interval between RT and surgery was 42 days for patients having complete pathological response or microscopic residual disease, compared with 33.5 days for patients with partial response or no change ($P = 0.15$). For patients undergoing LAR, the mean pathologic distal free margin was 2.2 cm (standard deviation (s.d.) 1.24, range 0.3–4 cm). Surgical margins were negative in 49 patients, positive in one and uncertain in three.

### Choice of surgical procedure

Indications regarding the type of surgical procedure foreseen was available in the clinical records of all patients prior to RT. APR was planned in 21 patients, a restorative procedure in seven patients, and in 25 patients the decision was intended to be made as a function of the tumour response to preoperative treatment. The final decision was taken by the surgeon in the immediate preoperative period according essentially to the tumour response. Table 2 displays the comparison of the planned type of surgery to the procedure actually performed. The mean distance from the anal verge to the tumour was 3 cm (s.d. 1.45, range 0.5–6 cm) and 4.5 cm (s.d. 1.25, range 1.5–6 cm) in patients operated by APR and LAR respectively ($P = 0.0007$).

### Toxicity

During preoperative therapy, 63% of patients presented with grade 1–2, 33% with grade 3 and one patient with a grade 4 acute toxicity. Acute complications involved the perineal skin in most cases, while diarrhoea, proctitis and urinary tract symptoms (dysuria, urgency, frequency) were commonly observed reactions. The grade 4 toxicity consisted of an acute small bowel obstruction managed conservatively, occurring at a dose of 25.2 Gy delivered by a 2-field technique in a patient receiving a continuous 5-FU infusion (treatment stopped definitively). One grade 3 leukopenia and one case of venous thrombosis were also documented.

Thirteen patients presented with post-operative complications: six in the APR group and seven in the conservation surgery group. There were two abdominal wound dehiscences (one treated surgically), three perineal and three abdominal wound abscesses, one colic ischaemia (treated surgically), one fistula after colostomy closing and three partial anastomotic disruptions (one managed surgically).

During follow-up, two patients from the restorative surgery group required surgical treatment for a late complication. One presented with small bowel obstruction managed surgically, and one presented with a presacral abscess requiring a definitive sigmoidostomy. Among 11 patients having had sphincter-saving surgery with a minimum of 1-year follow-up, the MSKCC anal function score was excellent in seven cases, good in two and fair in two.

© 2000 Cancer Research Campaign 

**Table 3** EORTC QLQ-C30 mean and median scale and single-item scores according to the type of surgery

| | APR group (11 patients) | | Restorative surgery group (12 patients) | | $P^a$ |
|---|---|---|---|---|---|
| | Means (s.d.) | Medians (range) | Means (s.d.) | Medians (range) | |
| Functional scales | | | | | |
| Physical function | 87 (22) | 100 (40–100) | 70 (34) | 80 (0–100) | 0.2 |
| Role function | 86 (19) | 83 (33–100) | 77 (32) | 100 (16–100) | 0.97 |
| Emotional function | 84 (24) | 91 (33–100) | 82 (21) | 91 (41–100) | 0.8 |
| Cognitive function | 88 (17) | 100 (50–100) | 85 (23) | 100 (33–100) | 0.95 |
| Social function | 85 (16) | 83 (66–100) | 78 (35) | 100 (0–100) | 0.87 |
| Global quality of life | 78 (14) | 83 (58–100) | 67 (25) | 66 (33–100) | 0.38 |
| Symptom scales | | | | | |
| Fatigue | 24 (33) | 11 (0–100) | 25 (25) | 16 (0–66) | 0.68 |
| Pain | 10 (13) | 0 (0–33) | 18 (22) | 16 (0–66) | 0.49 |
| Nausea and vomiting | 1.5 (5) | 0 (0–16) | 3 (9) | 0 (0–33) | 0.99 |
| Single items | | | | | |
| Dyspnoea | 6 (13) | 0 (0–33) | 14 (22) | 0 (0–66) | 0.49 |
| Sleep disturbance | 18 (17) | 33 (0–33) | 14 (33) | 0 (0–100) | 0.24 |
| Appetite loss | 6 (13) | 0 (0–33) | 3 (9) | 0 (0–33) | 0.68 |
| Diarrhoea | 9 (15) | 0 (0–33) | 16 (26) | 0 (0–66) | 0.66 |
| Constipation | 0 (0) | 0 (0–0) | 22 (29) | 0 (0–66) | 0.09 |
| Financial impact | 18 (34) | 0 (0–100) | 19 (36) | 0 (0–100) | 0.97 |

APR, abdomino-perineal resection; s.d., standard deviations; [a]*P*-value refers to the Mann–Whitney *U*-tests testing for differences in medians (the means are given for informative purposes).

### Oncologic results

With a median follow-up of 23 months (range 4–74 months) from the start of RT, nine patients have died: five of rectal cancer, two of a second malignancy, one from intercurrent disease and one from unknown cause. The 3-year actuarial survival for all patients was 71.4% (95% confidence interval (CI) 53–89%).

During follow-up, 15 patients presented with disease progression. Locoregional failures were observed in seven patients, either alone (four) or with distant metastasis (three). Distant metastasis alone occurred in nine patients, involving either the lung (seven) or the liver (five). The 3-year actuarial locoregional control rates were 77.5% (95% CI 61–93) for all patients, 100% for the 12 patients with marked pathological tumour response to RT, and 73% for the remaining patients. The 3-year disease-free survival rates were 58% (95% CI 40–75) for all patients, 89% for patients with pathological marked response and 52% for the remaining patients ($P$ = 0.16). According to pathological classification, 3-year disease-free survival rates were 76% and 47% for patients with pT0–2 and pT3–4 tumours respectively ($P$ = 0.057). No significant difference in disease-free survival was observed between patients operated by APR or by restorative surgery (Figure 1), or between patients receiving preoperative radiotherapy alone and those treated with radiochemotherapy.

### QOL outcomes

Twenty-six patients satisfied the inclusion criteria. All patients were contacted by telephone to solicit their participation. Twenty-three patients (88%) gave their approval to participate in the study, two refused and one was judged ineligible because of serious co-morbidities. Among the 23 participating patients, 11 were treated with APR and 12 with sphincter-sparing surgery. There were 17 males and six females. Age at the time of analysis, the median follow-up time, gender and performance status were similar in the two surgical groups.

### EORTC QLQ-C30 scores

The median and mean scale scores according to the type of surgery are given in Table 3. For all scale scores, no significant differences in medians were observed between the two surgical groups. Patients having undergone restorative surgery tended to report higher levels of constipation ($P$ = 0.09). Although patients having had APR seem to have more sleep disturbances, they tended to report higher levels of physical functioning and global QOL than did patients having had restorative resections.

### EORTC QLQ-CR38 scores

The general results for the two surgical groups are given in Table 4. No significant differences in median scores were observed between the two surgical groups for any of the scales. However, APR group patients tended to report a lower body image score ($P$ = 0.12) but a higher future perspective score. The sexual functioning score was very low in both groups. In males, sexual dysfunction symptom score was higher in the APR group. None of females responded to questions concerning the sexual dysfunction scale. The symptom score related to anorectal function in patients having had restorative surgery was comparable to the symptom score related to the stoma in the subgroup having had APR.

### DISCUSSION

In solid tumour oncology the past two decades have been marked by the success of multimodality treatment programmes aiming at respecting body integrity and attempting to preserve organ function. For cancers of the distal rectum the development of such therapeutic approaches in many centres has already resulted in a restriction in the indications for APR. While restorative surgical procedures have long been proposed for highly selected superficial lesions, the availability of sophisticated bowel stapling techniques has allowed restorative procedures to be applied to a much wider spectrum of

 © 2000 Cancer Research Campaign

**Table 4** EORTC QLQ-CR38 mean and median functional scale and symptom scores according to the type of surgery

| Scales | No. patients APR/Restorative | APR group | | Restorative surgery group | | P[a] |
|---|---|---|---|---|---|---|
| Functional scales | | Means (s.d.) | Medians (range) | Means (s.d.) | Medians (range) | |
| Body image | 11/12 | 72 (25) | 77 (33–100) | 87 (25) | 100 (33–100) | 0.12 |
| Future perspective | 10/12 | 80 (23) | 83 (33–100) | 66 (31) | 66 (0–100) | 0.35 |
| Sexual functioning | 11/11 | 27 (32) | 16 (0–100) | 18 (20) | 16 (0–66) | 0.71 |
| Sexual enjoyment | 5/7 | 60 (28) | 66 (33–100) | 47 (32) | 33 (0–100) | 0.51 |
| Symptom scales | | | | | | |
| RT side-effects on micturition | 11/12 | 20 (25) | 11 (0–66) | 23 (22) | 22 (0–66) | 0.68 |
| General gastro-intestinal | 11/12 | 12 (10) | 13 (0–33) | 15 (13) | 13 (0–46) | 0.53 |
| Defecation problems | –/12 | – | – | 17 (15) | 14 (0–52) | – |
| Stoma-related problems | 11/– | 20 (22) | 14 (0–76) | – | – | – |
| Sexual dysfuntion of males | 8/6 | 50 (30) | 50 (0–100) | 33 (27) | 25 (0–66) | 0.33 |
| Sexual dysfuntion of females | 0/0 | – | – | – | – | – |
| Weight loss | 11/12 | 0 (0) | 0 (0–0) | 8 (20) | 0 (0–66) | 0.49 |

APR, abdomino-perineal resection; s.d., standard deviations; [a]*P*-value refers to the Mann–Whitney *U*-tests testing for differences in medians (the means are given for informative purposes).

patients with rectal cancers (Papillon and Gerard, 1990; Marks et al, 1993). Preoperative RT can potentially increase the feasibility of sphincter-saving resections by reducing the tumour volume and by sterilizing local tumour extensions, compensating thereby for the very narrow lateral and caudal resection margins (Papillon and Gerard, 1990; Marks et al, 1993; Rouanet, 1995; Allal, 1996). The apparently satisfactory oncological results thus obtained, especially when viewed in the light of QOL end points, has led to the increasing use of multimodal restorative approaches.

To assess the incorporation of these therapeutic strategies into our routine practice, we analysed the choice of surgical procedures, oncological results and QOL outcomes in a recent series of 53 patients with cancers involving the distal rectum, the large majority of whom would have been submitted to APR little more than a decade ago (Williams et al, 1983a; Billingham, 1992). We found that 24 patients (45%) were able to undergo restorative surgical procedures, in 20 cases using LAR with coloanal anastomosis. Moreover, in 25 patients (47%) the decision regarding the type of surgery was not taken at initial surgical consultation, but rather was explicitly based upon the quality of tumour response to preoperative therapy. The proportion of sphincter-saving procedures appears low compared with the 76–83% rates reported in prospective studies of this approach (Minsky et al, 1995; Rouanet et al, 1995; Maghfoor et al, 1997). However, this apparent discrepancy may be explained by differences in patient selection, particularly regarding tumour stage and distance from the anal verge. Indeed, in the series of Minsky et al (1995) and Rouanet et al (1995), no patients had T4 tumours or lesions extending below 2.7 cm, whereas in the present series 32% of patients presented clinically with T4 tumours and 28% of patients had tumours extending within 2.7 cm from the anal verge. Our findings are consistent with the 50% rate of restorative resections reported in a large prospective trial studying the value of preoperative therapy in unselected patients with rectal carcinomas (Hyams et al, 1997).

It is likely in most cases that the distance between the anal verge and the lower edge of the tumour continues to dictate the choice of surgical technique. In the APR group the mean distance between tumour and anal verge was significantly smaller than that of the restorative surgery group (3 cm vs 4.5 cm). Based on operative series, 2 cm is widely considered the minimum distal safety margin (Polett and Nicholls, 1983; Williams et al, 1983a). In our patients operated by LAR, the mean distal surgical margin was 2.2 cm, with 11 (48%) patients having had a safety margin of less than 2 cm (min 0.3 cm). It is apparent that any increase in the proportion of restorative procedures could only occur at the expense of diminishing distal surgical margins. It is noteworthy that similar outcomes have been reported for patients with < 2 cm and those with >2 cm safety margins (Polett and Nicholls, 1983), and it is not excluded that a narrower surgical margin will come to be considered oncologically acceptable following preoperative RT. The validity of this notion is already suggested by some series reporting good locoregional control with mean margins of 1.5 cm (Rouanet et al, 1995), or for tumours located in the distal 2 cm of the rectum (Mohiuddin et al, 1998). In the other hand, the quality of the circumferential surgical margin is known to be crucial for the oncological outcome (Quirke et al, 1986). However, the minimum safe free lateral margin remains unknown (Heald and Karanjia, 1992), particularly in conjunction with preoperative RT.

Considering the relatively advanced stage of patients in the present series, the 3-year locoregional control rate of 77.5% can be considered as satisfactory. Only two of 24 patients (8%) failed locally after restorative resections, consistent with the results of reported series using preoperative RT (Marks et al, 1993; Minsky et al, 1995; Rouanet et al, 1995). The relatively low disease-free survival rate (58%) reflects the rather unfavourable initial disease presentation, with three patients already having distant metastases at time of surgery. No significant differences in outcomes were observed between the two surgical groups. However, it is noteworthy that patients presenting with marked pathological tumour response, with absent or only microscopic residual cancer after preoperative therapy, had a more favourable outcome. No locoregional recurrence and only one distant metastasis (8%) was observed in these 12 patients, compared with seven locoregional failures (17%) and 14 metastases (34%) in tumours showing no change or only partial response. Thus, as suggested by others (Berger et al, 1997), post-RT down-staging may have a certain prognostic value. Whether the improved outcome can be attributed to the effect of preoperative therapy or to particular clinical or biological characteristics of the responding tumours remains speculative.

*© 2000 Cancer Research Campaign*

Despite certain weaknesses that are inherent to the retrospective design of the present study (different RT techniques, different chemotherapy regimens) acute tolerance of preoperative treatment appeared acceptable in our hands, with all patients but one completing the planned programme. The single patient developing partial small bowel obstruction during preoperative therapy was receiving continuous 5-FU chemotherapy and was treated with opposed pelvic fields, a technique that is known to irradiate unacceptably large normal tissue volumes. This latter technique was used in the initial period of the study in one institution and was changed definitively after. Otherwise the 33% rate of grade 3 acute reactions is similar to those reported in the literature (Grann et al, 1997). In the perioperative period no fatal complication was observed, whilst partial anastomotic disruption was observed in three patients. Whether these latter complications were related to the preoperative RT remains speculative (Friedmann et al, 1987). Only one patient needed a permanent stoma for a late complication after restorative surgery. Similar rates of post-operative complications were reported in other series (Minsky et al, 1995; Rouanet et al, 1995).

We were able to assess anal sphincter function in 11 patients having undergone restorative resections and alive without recurrence at a minimum follow-up of 1 year. According to the MSKCC score, function was good to excellent in 9/11 patients (82%), whilst two patients had fair function. These results are similar to those of Minsky et al (1995) and Rouanet et al (1995), who reported 77% good to excellent and 71% perfect anal function respectively. However, a large number of evaluable patients and longer follow-up are needed to obtain a more precise notion of anal function in this subset of patients. It is noteworthy that prospective trials have shown a higher rate of long-term anorectal dysfunction in patients treated with either preoperative (Dahlberg et al, 1998) or post-operative RT (Lundby et al, 1997) compared with patients treated with surgery alone. Moreover, results can vary widely according to the scoring system and the use of subjective or objective tools (Lewis et al, 1995).

It is generally believed that patients having had sphincter-conserving procedures enjoy better QOL outcomes than patients with APR (Williams and Johnston, 1983; Marquis et al, 1992). In a review of 17 studies assessing QOL in colorectal cancer patients, both those with and those without stomas reported impairment in the four QOL domains studied (physical, psychological, social and sexual), whilst the latter domain was more affected in stoma patients (Sprangers et al, 1995). However, the magnitude of the differences in the scores, particularly in the sexual domain, varied widely, and the results were sometimes inconsistent. Moreover, the lack of a prospective study design, the clear difference in patient characteristics, particularly regarding age and the sphincter-conserving procedures used, may prevent a direct extrapolation of these findings to patients with coloanal anastomoses after preoperative RT.

Aware of the modest size of our study, we tried to assess QOL outcomes in the two surgical groups of patients. By using the cancer-specific EORTC-QLQ-C30 questionnaire, no significant differences in median scores were observed in any of the function scales studied (physical, role, social, emotional and cognitive functions, as well as overall QOL). Interestingly some scores, such as physical and overall QOL scores, were even higher in the APR group. This finding seems to be in contradiction with the results of some authors (Frigell et al, 1990; Williams and Johnston, 1983; MacDonald and Anderson, 1985), who reported better general QOL and physical activity in non-stoma patients, while general state of health was reported to be similar in another large series (Wirsching et al, 1975). This finding can be explained partially by the fact that, unlike many other series, the two groups of patients were equally distributed according to age, WHO performance status, gender, and median follow-up, all of which are important parameters when assessing QOL outcomes. However, one should note that only five of 12 patients in the restorative surgery group had a J pouch, a technical aspect that is known to increase the functional outcome in this subset of patients. For the symptom scales, whilst non-significant, the restorative group tended to report higher levels of constipation and pain symptoms, whereas the APR group tended to report more sleeping disturbance. Others authors also find more frequent complaints of constipation in non-stoma patients (Williams and Johnston, 1983; MacDonald and Anderson, 1985), and more sleeping disturbances in stoma patients were noted in another series (Frigell et al, 1990).

The site-specific EORTC-QLQ-CR38 questionnaire confirmed the findings of others (Williams and Johnston, 1983; MacDonald and Anderson, 1985) that APR was associated with a lower body image score and a higher sexual dysfunction score in males, although statistical significance could not be demonstrated in this small study. Moreover, it is noteworthy that the sexual functioning score was very low for both surgical groups, and that no differences were apparent in the sexual enjoyment scale. Development of nerve-sparing surgical procedures may play an important role in preventing such complications (Maas et al, 1998). No data could be provided on sexual dysfunction in females, since no women chose to answer the proposed questions. Problems of compliance have been reported in previous studies, especially when questions concerned sexual aspects of QOL (Klee et al, 1997). Finally, patients in the APR group tended to report a higher score for future perspective scale; this is thought to reflect a decreased concern about their future state of health. It is possible that after APR patients are more likely to believe that all the potential area of recurrence have been removed, leading to a greater sense of security.

In conclusion, this study reinforces the notion of the feasibility in routine practice of sphincter-saving surgery after preoperative RT with or without chemotherapy for cancers of the distal rectum. Preoperative therapy was associated with manageable toxicities, perioperative complications were acceptable, and the oncological results in patients having restorative resections appear similar to those obtained with APR. Any further increase in the proportion of sphincter-saving procedures will require a decrease in the minimal acceptable distal safety margins. This solution may be feasible after preoperative RT, particularly for good responders. Except for body image and sexual aspects in men, the other dimensions of QOL in our study were not necessarily better in the restorative surgery group at 1-year post treatment. However, the small number of patients studied disallows any formal conclusions, but rather suggests hypotheses to be studied prospectively. Thus, a well-designed prospective study would be required to firmly establish the superiority of restorative procedures regarding QOL aspects in cancers of the distal rectum.

### REFERENCES

Aaronson NK, Ahmedzai S, Bergman B, Bullinger M, Cull A, Duez NJ, Filiberti A, Flechtner H, Fleishman SB and de Haes JC (1993) The European Organization for Research and Treatment of Cancer QLQ-C30: a quality-of-life instrument for use in international clinical trials in oncology. *Natl Cancer Inst* **85**: 365–376

*© 2000 Cancer Research Campaign*

Allal AS (1996) Cancer du bas rectum: vers une approche thérapeutique integrant la radiothérapie. *Bull Cancer* **83**: 189–196

Berger C, de Muret A, Garaud P, Chapet S, Bourlier P, Reynaud-Bougnoux A, Dorval E, de Calan L, Huten N, le Floch O and Calais G (1997) Preoperative radiotherapy (RT) for rectal cancer: predictive factors of tumor downstaging and residual tumor cell density (RTCD): prognostic implications. *Int J Radiat Oncol Biol Phys* **37**: 619–627

Billingham RP (1992) Conservative treatment of rectal cancer. *Cancer* 70: 1355–1363

Dahlberg M, Glemelius B, Graf W and Pahlman L (1998) Preoperative irradiation affects functional results after surgery for rectal cancer: results from a randomized study. *Dis Colon Rectum* **41**: 543–549

Friedmann P, Garb JL, McCabe DP, Chabot JR, Park WC, Stark A, Coe NP and Page DW (1987) Intestinal anastomosis after preoperative radiation therapy for carcinoma of the rectum. *Surg Gynecol Obstet* **164**: 257–260

Frigell A, Ottander M, Stenbeck H and Pahlman L (1990) Quality of life of patients treated with abdominoperineal resection or anterior resection for rectal carcinoma. *Ann Chir Gynaecol* **79**: 26–30

Grann A, Minsky BD, Cohen AM, Saltz L, Guillem JG, Paty PB, Kelsen DP, Kemeny N, Ilson D and Bass-Loeb J (1997) Preliminary results of preoperative 5-fluorouracil, low-dose leucovorin, and concurrent radiation therapy for clinically resectable T3 rectal cancer. *Dis Colon Rectum* **40**: 515–522

Heald RJ and Karanjia ND (1992) Results of radical surgery for rectal cancer. *World J Surg* **16**: 848–857

Heald RJ and Ryall RD (1986) Recurrence and survival after total mesorectal excision for rectal cancer. *Lancet* **1**: 1479–1482

Hyams DM, Mamounas EP, Petrelli N, Rockette H, Jones J, Wieand HS, Deutsch M, Wickerham L, Fisher B and Wolmark N (1997) A clinical trial to evaluate the worth of preoperative multimodality therapy in patients with operable carcinoma of the rectum: a progress report of National Surgical Breast and Bowel Project Protocol R-03. *Dis Colon Rectum* **40**: 131–139

Kaplan EL and Meier P (1958) Non-parametric estimation from incomplete observations. *J Am Stat Assoc* **53**: 457–481

Klee M, Groenvold M and Machin D (1997) Quality of life of Danish women: population-based norms of the EORTC QLQ-C30. *Qual Life Res* **6**: 27–34

Lewis WG, Williamson ME, Kuzu A, Stephenson BM, Holdsworth PJ, Finan PJ, Ash D and Johnston D (1995) Potential disadvantages of post-operative adjuvant radiotherapy after anterior resection for rectal cancer: a pilot study of sphincter function, rectal capacity and clinical outcome. *Int J Colorectal Dis* **10**: 133–137

Lundby L, Jensen VJ, Overgaard J and Laurberg S (1997) Long-term colorectal function after postoperative radiotherapy for colorectal cancer [letter]. *Lancet* **350**: 564

MacDonald LD and Anderson AR (1985) The health of rectal cancer patients in the community. *Eur J Surg Oncol* **11**: 235–241

Maas CP, Moriya Y, Steup WH, Kiebert GM, Kranenbarg WM and van de Velde CJ (1998) Radical and nerve-preserving surgery for rectal cancer in The Netherlands: a prospective study on morbidity and functional outcome. *Br J Surg* **85**: 92–97

Maghfoor I, Wilkes J, Kuvshinoff B, Westgate S, Bryer M and Perry MC (1997) Neoadjuvant chemoradiotherapy with sphincter-sparing surgery for low-lying rectal cancer. Proceedings of ASCO. *J Clin Oncol* **16**: 274(Abstract)

Marks G, Mohiuddin M and Masoni L (1993) The reality of radical sphincter preservation surgery for cancer of the distal 3 cm of rectum following high-dose radiation. *Int J Radiat Oncol Biol Phys* **27**: 779–783

Marquis R, Lasry JC, Heppell J, Potvin C, Falardeau M and Robidoux A (1992) Quality of life of patients after restorative surgery for cancer of the rectum. *Ann Chir* **46**: 830–838

Minsky BD, Cohen AM, Enker WE and Paty P (1995) Sphincter preservation with preoperative radiation therapy and coloanal anastomosis. *Int J Radiat Oncol Biol Phys* **31**: 553–559

Mohiuddin M, Regine WF, Marks GJ and Marks JW (1998) High-dose preoperative radiation and the challenge of sphincter-preservation surgery for cancer of the distal 2 cm of the rectum. *Int J Radiat Oncol Biol Phys* **40**: 569–574

Papillon J and Gérard JP (1990) Role of radiation therapy in anal preservation for cancers of the lower third of the rectum. *Int J Radiat Oncol Biol Phys* **19**: 1219–1220

Perez CA and Brady LW (1992) Overview. In: *Principles and Practice of Radiation Oncology*, 2nd edn, Perez CA and Brady LW (eds), pp. 51–55. Lippincott: Philadelphia

Pollett WG and Nicholls RJ (1983) The relationship between the extent of distal clearance and survival and local recurrence rates after curative anterior resection for carcinoma of the rectum. *Ann Surg* **198**: 159–163

Quirke P, Durdey P, Dixon MF and Williams NS (1986) Local recurrence of rectal adenocarcinoma due to inadequate surgical resection. Histological study of lateral tumour spread and surgical excision. *Lancet* **2**: 996–999

Rouanet P, Fabre JM, Dubois JB, Dravet F, Saint Aubert B, Pradel J, Ychou M, Solassol C and Pujol H (1995) Conservative surgery for low rectal carcinoma after high-dose radiation. Functional and oncologic results. *Ann Surg* **221**: 67–73

Sprangers MA, Taal BG, Aaronson NK and te Velde A (1995) Quality of life in colorectal cancer. Stoma vs. nonstoma patients. *Dis Colon Rectum* **38**: 361–369

Sprangers MAG, te Velde A and Aaronson NK, on behalf of the European Organization for Research and Treatment of Cancer Study Group on Quality of Life (1999) The construction and testing of the EORTC Colorectal Cancer Specific Quality-of-Life questionnaire Module (the QLQ-CR38). *Eur J Cancer* (in press)

Williams NS, Dixon MF and Johnston D (1983) Reappraisal of the 5 centimetre rule of distal excision for carcinoma of the rectum: a study of distal intramural spread and of patients' survival. *Br J Surg* **70**: 150–154

Williams NS and Johnston D (1983) The quality of life after rectal excision for low rectal cancer. *Br J Surg* **70**: 460–462

Wirsching M, Druner HU and Herrmann G (1975) Results of psychosocial adjustment to long-term colostomy. *Psychother Psychosom* **26**: 245–256

*© 2000 Cancer Research Campaign*
